# Optimal crystalloid volume ratio for blood replacement for maintaining hemodynamic stability and lung function: an experimental randomized controlled study

**DOI:** 10.1186/s12871-019-0691-0

**Published:** 2019-02-13

**Authors:** Gergely H. Fodor, Walid Habre, Adam L. Balogh, Roberta Südy, Barna Babik, Ferenc Peták

**Affiliations:** 1 Department of Medical Physics and Informatics, University of Szeged, 9 Koranyi fasor, Szeged, H-6720 Hungary; 2 Unit for Anesthesiological Investigations, Department of Anesthesiology, Pharmacology and Intensive Care, University Hospitals of Geneva, University of Geneva, 1 Rue Michel Servet, CH-1205 Geneva, Switzerland; 3 Department of Anesthesiology and Intensive Therapy, University of Szeged, 8 Semmelweis str, Szeged, H-6725 Hungary

**Keywords:** Fluid resuscitation, Respiratory mechanics, Pulmonary edema, Lung function, Fluid replacement

## Abstract

**Background:**

Crystalloids are first line in fluid resuscitation therapy, however there is a lack of evidence-based recommendations on the volume to be administered. Therefore, we aimed at comparing the systemic hemodynamic and respiratory effects of volume replacement therapy with a 1:1 ratio to the historical 1:3 ratio.

**Methods:**

Anesthetized, ventilated rats randomly included in 3 groups: blood withdrawal and replacement with crystalloid in 1:1 ratio (Group 1, *n* = 11), traditional 1:3 ratio (Group 3, *n* = 12) and a control group with no interventions (Group C, *n* = 9). Arterial blood of 5% of the total blood volume was withdrawn 7 times, and replaced stepwise with different volume rations of Ringer’s acetate, according to group assignments. Airway resistance (Raw), respiratory tissue damping (G) and tissue elastance (H), mean arterial pressure (MAP) and heart rate (HR) were assessed following each step of fluid replacement with a crystalloid (CR1-CR6). Lung edema index was measured from histological samples.

**Results:**

Raw decreased in Groups 1 and 3 following CR3 (*p* < 0.02) without differences between the groups. H elevated in all groups (*p* < 0.02), with significantly higher changes in Group 3 compared to Groups C and 1 (both *p* = 0.03). No differences in MAP or HR were present between Groups 1 and 3. Lung edema was noted in Group 3 (*p* < 0.05).

**Conclusions:**

Fluid resuscitation therapy by administering a 1:1 blood replacement ratio revealed adequate compensation capacity and physiological homeostasis similar with no lung stiffening and pulmonary edema. Therefore, considering this ratio promotes the restrictive fluid administration in the presence of continuous and occult bleeding.

## Background

With the increased awareness of the potential harmful effect of colloids on the organs, crystalloids are considered as first line fluid resuscitation therapy. Moreover, goal-directed fluid therapy is now a well-established strategy in routine clinical practice in the perioperative period. The main feature of this strategy is to reduce the amount of fluid administered to the patients while maintaining optimal cardiac output and adequate organ perfusion [1, 2]. However, crystalloids have short hemodynamic beneficial effect due to the extravasation into the interstitial space [3] with a very low (less than 20%) intravascular volume effect [4].

There are various clinical situations where continuous and occult bleeding (such as liver surgery and trauma, transplants, scoliosis surgery, etc.) may occur and the clinicians may have to face higher ratio of fluid replacement to maintain hemodynamic stability. Despite the routine use of crystalloids in these clinical situations, the optimal volume to be administered to re-establish blood volume and maintain optimal cardiac output and end-organ perfusion is arbitrary. This ambiguity arises from the lack of evidence-based recommendations with regard to the volume of crystalloid solution to be administered in order to compensate for the same blood lost volume. Historically, 3- to 4-fold ratio is recommended in various textbooks as a traditional approach to counterweigh blood loss [5]. Despite the recent European guidelines to use a restricted volume replacement [6], closer to a 1:1 ratio, there have been no studies providing evidence for the choice of the optimal crystalloid volume to be administered to maintain hemodynamic stability with minimal adverse effect due to interstitial water extravasation.

We recently demonstrated that measurements of respiratory mechanics provide sensitive indices of lung water extravasation as a consequence of fluid replacement therapy in an experimental model of acute hemorrhage [7, 8]. Therefore, we aimed at using a similar approach to clarify the debate on the optimal crystalloid volume ratio following occult and continuous bleeding. We hypothesized that when compared to the historical ratio of 1:3, crystalloid fluid replacement in a 1:1 ratio is sufficient to ensure hemodynamic stability with little effect on lung function as a surrogate for interstitial edema development.

## Methods

### Ethics

The experimental procedures were approved by the institutional ethics committee for experimental research of the University of Szeged (no. I-74-50/2012) and by the National Food Chain Safety and Animal Health Directorate of Csongrád County, Hungary (no. XIV/152/2013). The procedures were performed according to the guidelines of the Scientific Committee of Animal Experimentation of the Hungarian Academy of Sciences (updated Law and Regulations on Animal Protection: 40/2013. (II. 14.) Government of Hungary), following the EU Directive 2010/63/EU on the protection of animals used for scientific purposes, and reported in compliance with the ARRIVE guidelines.

#### Animal preparations

Anesthesia of 32 male adult Wistar rats (330 [285–388] g from the animal facility of Faculty of Pharmacy, University of Szeged, Hungary) was induced with an intraperitoneal injection of sodium pentobarbital (45 mg/kg). Tracheostomy was performed after subcutaneous administration of lidocaine (2–4 mg/kg) and a polyethylene cannula was introduced into the trachea (16 G, B. Braun Melsungen AG, Melsungen, Germany). A small animal ventilator (Model 683, Harvard Apparatus, South Natick, MA, USA) was used to ventilate the animals in a supine position with room air (70 breaths/min, tidal volume 7 ml/kg) through the tracheal tube. The left femoral vein was cannulated (Abbocath 22 G) for drug delivery and fluid replacement. This line was used to maintain anesthesia by repeated injections of sodium pentobarbital (12 mg/kg, every 30 min) and muscle relaxation was achieved by regular administration of pipecuronium (0.1 mg/kg, every 30 min, Arduan, Richter-Gedeon, Budapest, Hungary). The left femoral artery was catheterized (Abbocath 22 G) and connected to a pressure transducer (Model TSD104A, Biopac, Santa Barbara, CA, USA) for continuous systemic blood pressure monitoring to assess mean arterial pressure (MAP), and to allow blood withdrawal, as part of the experimental protocol. A data collection and acquisition system (Biopac, Santa Barbara, CA, USA) was used to monitor the MAP, ECG and heart rate (HR) continuously. Body temperature was kept in the 37 ± 0.5 °C range by utilizing a heating pad.

#### Measurement of respiratory mechanical parameters

The forced oscillation technique was applied in short (6-s-long) end-expiratory pauses, as detailed previously [9, 10] to obtain the input impedance of the respiratory system (Zrs). Briefly, ventilation was ceased at end-expiration and the tracheal cannula was switched to a loudspeaker-in-box system by means of a three-way tap. A computer-generated small amplitude (< ±1 cmH_2_O) pseudorandom signal composed of 23 non-integer multiples ranging 0.5–20.75 Hz was delivered by the loudspeaker through a 100-cm-long, 2-mm-internal diameter polyethylene wave-tube. The lateral pressures were measured at the loudspeaker end (P_1_) and at the tracheal end (P_2_) of the wave-tube by using two identical pressure transducers (model 33NA002D, ICSensors, Milpitas, CA, USA). The signals P_1_ and P_2_ were low-pass filtered at 25 Hz, and digitized by a data acquisition board (NI USB-6211, National Instruments, Austin, TX) at a rate of 256 Hz. Pressure transfer functions (P_1_/P_2_) were calculated with fast Fourier transformation (4-s time windows and 95% overlapping) from each 6-s recordings. Zrs was calculated as the load impedance of the wave-tube [11]. The input impedance of the ET tube and the connections was also determined, and was subtracted from each Zrs spectrum.

A model containing a frequency-independent resistance (Raw) and inertance (Iaw) and a constant-phase tissue compartment consisting of tissue damping (G) and elastance (H) was fitted to the Zrs spectra by minimizing the weighted difference between the measured and the modelled impedance data [12]. G describes the dissipative (damping or resistive) properties, while H reflects stiffness (elastance) of the respiratory tissues. Raw and Iaw characterize mainly the resistance and inertance of the airways, since the contribution of the chest wall to these parameters in rats is minor [13].

#### Pulmonary edema assessments

At the end of the experimental protocol the animals were euthanized with an intravenous overdose of pentobarbital sodium (300 mg/kg), then a midline thoracotomy was performed. The right lungs were fixed by instilling 4% formaldehyde at a hydrostatic pressure of 20 cmH_2_O via the tracheal cannula. The lungs were excised in one piece and placed into 4% buffered formalin until further processing. After complete fixation, transhilar horizontal sections (perpendicular to the longitudinal axes of the lung from the hilum) were embedded in paraffin. Two 5-μm sections were prepared in each lung specimen and were stained with hematoxylin-eosin. Digitized images of 10 non-overlapping fields of view were used to obtain the edema index around randomly selected pulmonary vessels by dividing the lumen area by the total area of the pulmonary vessel (edema cuff area + vessel lumen area). Histological images were analyzed by the same investigator in a blind fashion and in a random sequence by using JMicroVision image analysis software (version 1.2.7).

Three-to-four tissue samples were dissected from the different lobes of the non-fixated left lungs; these samples were weighed to establish the wet-to-dry weight ratio (W/D) as an index of the lung water content.

#### Study protocol

**Fig. 1:**
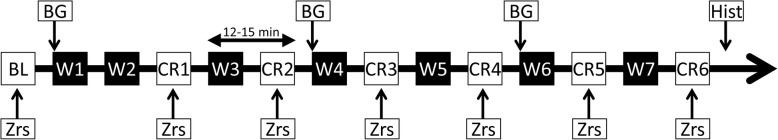
Experimental protocol. BL: baseline, W1-W7: blood withdrawal (5% of total blood volume), CR1-CR6: fluid replacement maneuvers, Zrs: a set of respiratory impedance measurements, BG: arterial blood gas measurements. Each withdrawal-replacement step lasted about 12–15 min, resulting in a total duration of the fluid resuscitation period of around 90 min

The rats were assigned to the protocol groups randomly and the same experimental protocol was applied to both treatment groups apart from the volume of replaced fluid (as depicted in Fig. 1). Following animal preparations and reaching a steady-state condition, a lung hyperinflation was applied by occluding the expiratory port of the ventilator for 1 respiratory cycle to standardize the lung volume history. Baseline respiratory mechanical parameters were determined by measuring 4 reproducible Zrs data sets. Blood loss was achieved by withdrawing 5% (approximately 1 ml) of total estimated blood volume (TBV) [14] via the femoral artery, then after 7 min, withdrawal of blood was repeated once again identically. Following these two withdrawals of arterial blood, fluid replacement was carried out by a rapid injection of Ringer’s acetate solution (Ringerfundin B. Braun, B. Braun Melsungen AG, Melsungen, Germany) into the femoral vein in accordance with the group allocation: Group 1 (*n* = 11) received 5% of TBV (1:1 ratio) and Group 3 (*n* = 12) received 15% of TBV (1:3 ratio) at each replacement. A set of Zrs data was recorded three minutes later. The blood withdrawal-replacement was repeated five more times (until a total of 7 withdrawal and 6 replacement steps) with additional Zrs recordings after each replacement maneuvers. Blood gas analyses were performed from the first, fourth and sixth withdrawn arterial blood samples (epoc Reader and Host, Epocal Inc., Ottawa, ON, Canada) to determine hematocrit (Hct), pH and partial pressures of oxygen (PaO_2_) and carbon dioxide (PaCO_2_). Arterial oxygen content (CaO_2_) of the blood was calculated based on the following equation: CaO_2_ = SaO_2_ x Hb × 1.39 + (PaO_2_ × 0.03) with hemoglobin estimated from the hematocrit level based on the formula Hb (g/dl) = Hct (%)/3 [15]. At the end of the experimental protocol the lungs were processed for assessment of pulmonary edema as detailed above. Each withdrawal-replacement step lasted about 12–15 min, resulting in a total duration of the fluid resuscitation period of around 90 min.

A control group (Group C, *n* = 9) was also used with identical timing of the measurements, but no withdrawal and replacement, apart from approximately 0.3 ml for each arterial blood gas sample at the same time as in the protocol groups.

#### Statistical analyses

Group mean averages with SE values are reported. Normality was checked with the Shapiro-Wilk test. Considering the mean values of the primary outcome, H (4800 cmH_2_O/l) and its variability (650 cmH_2_O/l) established in earlier studies in rats [7], sample size estimation revealed that 10 rats are necessary in each group to detect 20% difference in the three groups with 80% power at significance level of 0.05. Two-way repeated measures analysis of variance (ANOVA) was used with the variables group assessment and time to establish the effects of these factors on respiratory mechanical, blood gas and hemodynamic parameters. The Holm-Sidak multiple comparison procedure was employed to compare the mechanical, blood gas and hemodynamic parameters under different conditions versus controls of both group assessment (Group C) and time (BL or first blood gas measurement). One-way ANOVA with the factor group assessment was used to assess effects of fluid volume on edema indices and to assess differences of final responses (those obtained at CR6). The Holm-Sidak multiple comparison procedure was applied for post hoc tests. The statistical tests were performed with SigmaPlot statistical software package (Version 13, Systat Software, Inc. Chicago, IL, USA). In each test, a significance level of *p* < 0.05 was applied.

## Results

Body weights of the rats did not differ significantly between the groups (335 [285–386] g for Group C, 333 [285–388] g for Group 1 and 324 [285–380] g for Group 3).

**Fig. 2:**
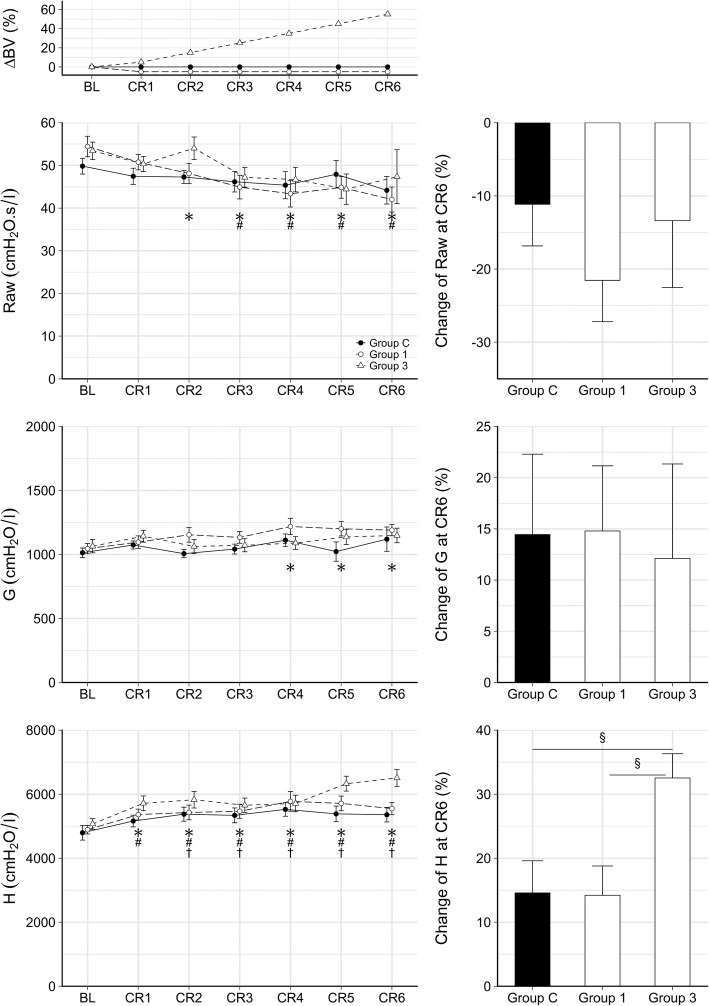
Mean (±SE) values of respiratory mechanical parameters during the experimental protocol. ΔBV: changes of total blood volume. The absolute values of airway resistance (Raw), tissue damping (G) and tissue elastance (H) over the experimental protocol (left) and their changes following the last fluid replacement maneuver relative to their corresponding baseline (right). BL: baseline. CR1-CR6: replacement maneuvers. *: *p* < 0.05 in Group 1 vs BL, #: *p* < 0.05 in Group 3 vs BL, †: *p* < 0.05 in Group C vs BL. §: *p* < 0.05 in the relative changes

Changes in respiratory mechanical parameters following each fluid replacement maneuver are demonstrated in Fig. 2. No significant changes in Raw were observed in Group C. While significant decreases in Raw were observed in Groups 1 (CR2–CR6, each *p* < 0.02) and 3 (CR3–CR6, each *p* < 0.02), there was no evidence for a difference in the relative changes in Raw between the groups. No significant changes were observed in G in any of the groups apart from slight increases from CR4 in Group 1 (CR4–CR6, each *p* < 0.03). Respiratory elastance worsened in all groups with mild changes in groups C and 1 (CR2–CR6, each *p* < 0.01 and CR1–CR6, each *p* < 0.01 for group C and group 1, respectively), whereas a significantly more severe deterioration was observed with time in group 3 (CR1–CR6, each *p* < 0.03).

Parameters assessed from arterial blood gas analyses are demonstrated in Table 1. The study protocol led to a significant decrease in Hct in both Groups 1 (*p* < 0.001 at W4 and W6) and 3 (*p* < 0.001 at W4 and W6). However, in comparison to Group C and 1, fluid replacement with a 1:3 ratio resulted in significantly lower values of Hct from the fourth blood withdrawal maneuver (W4, *p* < 0.001), implying lowered Hct in Group 3 after the third fluid replacement maneuver (CR3). Slight decrements of pH were obtained in Group 1 (*p* = 0.019 and *p* < 0.001 at W4 and W6, respectively) and Group 3 (*p* = 0.049 and *p* < 0.001 at W4 and W6, respectively) during the fluid replacement protocol, however no significant differences were detected between the groups. No significant changes were detected in PaO_2_ and PaCO_2_ in any of the protocol groups. Significant decreases were observed in CaO_2_ both in Group 1 and Group 3 at fourth (W4) and sixth (W6) blood withdrawal maneuvers (*p* < 0.001 for all) with significantly lower values in Group 3 compared to Group C at W6 (*p* = 0.004).

**Table 1:** Mean ± SE values of parameters obtained from arterial blood gas measurements

		W1	W4	W6
Hematocrit (%)	Group C	35.60 ± 0.77	34.24 ± 1.29	33.04 ± 1.88
	Group 1	36.64 ± 0.72	32.26 ± 0.81*	29.63 ± 1.23^*$^
	Group 3	36.39 ± 0.77	30.30 ± 1.09^*$^	27.09 ± 0.60^*$§^
pH	Group C	7.50 ± 0.01	7.45 ± 0.01	7.45 ± 0.03
	Group 1	7.52 ± 0.02	7.45 ± 0.03*	7.41 ± 0.02*
	Group 3	7.54 ± 0.02	7.47 ± 0.02*	7.41 ± 0.03*
PaO_2_ (mmHg)	Group C	70.75 ± 2.10	63.21 ± 2.86	67.35 ± 1.73
	Group 1	74.24 ± 2.84	69.06 ± 3.55	75.43 ± 3.22
	Group 3	75.28 ± 2.55	67.00 ± 4.10	71.06 ± 1.15
PaCO_2_ (mmHg)	Group C	30.51 ± 1.74	30.63 ± 1.57	27.57 ± 3.08
	Group 1	30.08 ± 1.55	31.07 ± 1.34	31.21 ± 1.49
	Group 3	27.94 ± 0.97	29.71 ± 0.97	23.52 ± 2.34
CaO_2_ (ml O_2_/100 ml)	Group C	15.67 ± 0.38	14.36 ± 0.56	14.37 ± 0.94
	Group 1	16.29 ± 0.35	14.00 ± 0.36*	12.97 ± 0.48*
	Group 3	16.21 ± 0.43	12.91 ± 0.48*	11.78 ± 0.22^*$^

*PaO*_2_ and *PaCO*_2_: arterial partial pressures of O_2_ and CO_2_, respectively. *CaO*_2_: arterial oxygen content. *W* blood withdrawal.*: *p*<0.05 vs. W1, $: *p*<0.05 vs. Group C, §: *p*<0.05 vs. Group 1.

**Fig. 3:**
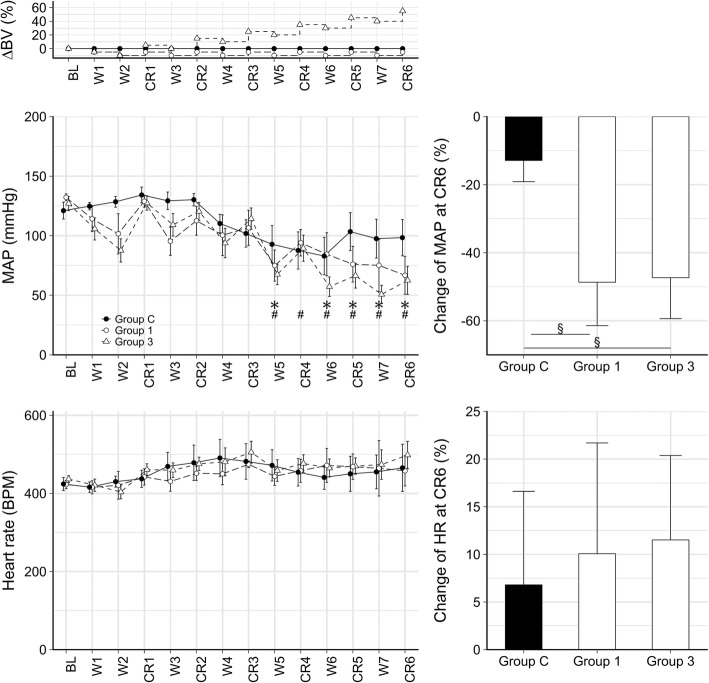
Mean (±SE) values of hemodynamic parameters during the experimental protocol (both at replacement and withdrawal steps). ΔBV: changes of total blood volume. Absolute values of mean arterial pressure (MAP) and heart rate (HR) over the experimental protocol (left) and their changes following the last replacement compared to baseline (right). BL: baseline. CR1-CR6: replacement maneuvers, W1-W7: blood withdrawal steps. *: *p* < 0.05 in Group 1 vs BL, #: *p* < 0.05 in Group 3 vs BL. §: *p* < 0.05 in the relative changes

Figure 3 exhibits the changes of systemic hemodynamic parameters. No significant changes in MAP or HR were detected in Group C, however, significantly lower values of MAP were observed in Group 1 and Group 3 from the fifth blood withdrawal (W5–CR6, each *p* < 0.05). The relative decreases in MAP at the end of the protocol were significantly greater in both Group 1 and Group 3 than those observed in Group C (*p* = 0.03 for both). No significant changes of HR were detected during the protocol.

**Fig. 4:**
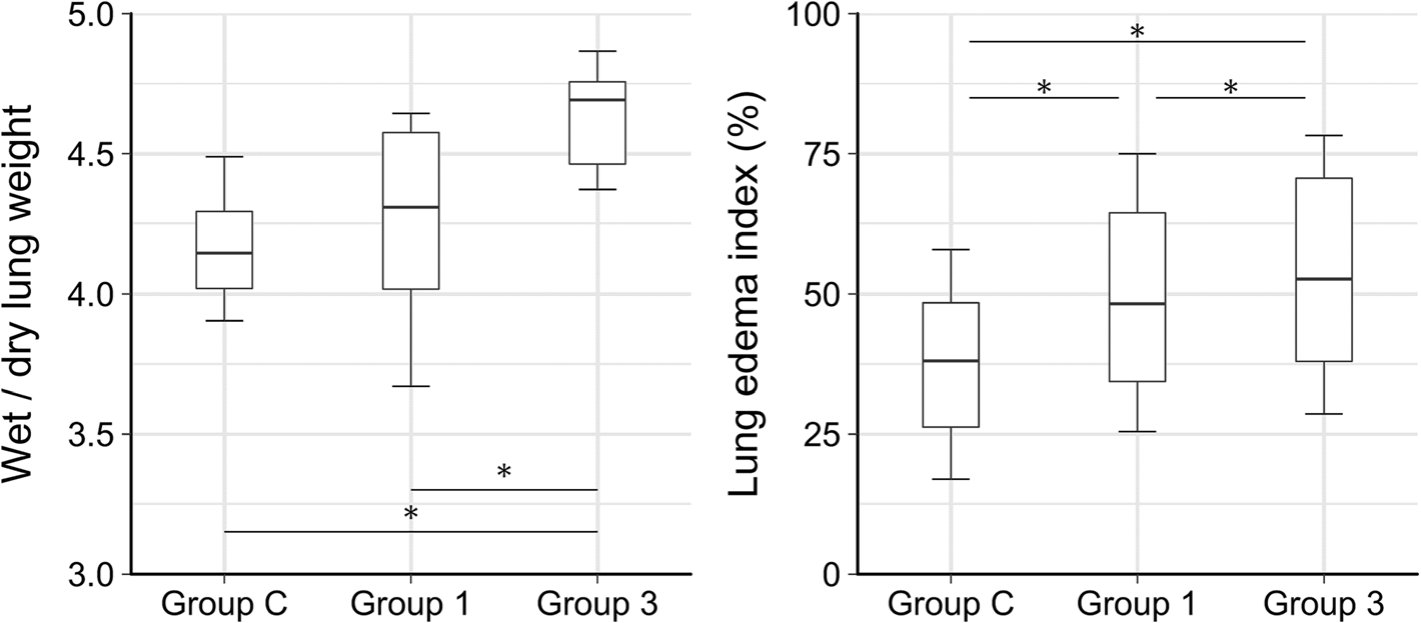
Edema index obtained by ratio of perivascular edema cuff and vascular area from the histological samples. *: *p* < 0.05

The results of lung edema assessments obtained from the protocol groups are summarized in Fig. 4. Wet-to-dry lung weight ratio was significantly greater in Group 3 than those obtained in Groups C (*p* = 0.05) and 1 (*p* = 0.048). Histological lung edema index was significantly elevated in Group 1 compared to Group C (*p* < 0.001), with even more severe elevations observed in Group 3 compared to Group 1 (*p* = 0.05).

## Discussion

We demonstrated in the present experimental study that compensating blood loss with crystalloids in a ratio of 1:1 led to lack of adverse alterations in the lung with maintenance of physiological homeostasis. Conversely, the use of a historical ratio of 1:3 resulted in a deterioration in respiratory tissue stiffness as a consequence of perivascular edema development. Moreover, fluid replacement in a ratio of 1:3 induced greater hemodilution as expressed in the significant decrease in the oxygen content of the blood.

In agreements with our previous study when the blood loss was replaced with normal saline [7], a slight decrease was observed in Raw in Group 1 and Group 3 following fluid resuscitations. While this bronchodilation is presumably caused by a catecholamine-release induced by a loss of blood volume [8], the lack of differences at the final replacement maneuver suggests that this catecholamine-release is counteracted by the restoration of vascular filling with crystalloid, which propagates the recovery of bronchial geometry.

The gradual increases in the respiratory tissue stiffness manifested by the elevations of H in Group C can be most probably attributed to a slight formation of atelectatic areas in the dependent lung regions subsequent to general anesthesia and supine position leading to lung volume loss [16]. Interestingly, fluid replacement with crystalloid at 1:1 ratio resulted in a similar trend in H, indicating that such intervention has no deleterious consequences in the lung tissue mechanics. In contrast with these mild changes, fluid replacement therapy with the larger crystalloid volume (1:3) led to severe respiratory tissue stiffening when the circulatory blood volume was increased above 140% of baseline. These adverse respiratory mechanical changes can be attributed to a significant increase in the pulmonary edema extent subsequent to severe hemodilution manifested in the significant drop in Hct level. The excessive edema development in the animals of Group 3 may lead ultimately to deterioration in gas exchange [17–19], and may be a risk factor for the development of acute respiratory distress syndrome [20]. Hypoxemia was not observed in the present study due to the relatively limited drop in CaO_2_ and Hct that allowed the maintenance of physiological homeostasis and the short duration of the experiment (90 min).

The mechanisms responsible for the changes in the respiratory mechanics following hemodilution are still to be fully characterized. In an earlier in vitro model, we demonstrated that decreased hematocrit values resulted in no change of Raw and decreases of G and H [21]. The contradiction between the previous in vitro and the current in vivo results suggest that although hematocrit content and blood viscosity affect the mechanical properties of the respiratory system, our present findings are governed mainly by systemic factors, such as the systemic catecholamine-release subsequent to blood loss [8].

There is still no clear consensus on the optimal fluid replacement therapy. Colloids have been used as volume-sparing agents for large-scale fluid losses [22], however due to the recent controversies related to hydroxyethyl-starch [23, 24] and albumin [24, 25], crystalloids are still highly recommended for fluid resuscitation [6, 26]. In clinical practice, not only the type of the applied solution, but the applied volume is also under debate. It is commonly accepted that although crystalloids are recommended in a goal-directed manner [27], these solutions are still applied in higher volumes compared to colloids [28]. There is increasing evidence for the benefit of intravenous fluid restriction during the perioperative period on patient recovery and outcome [29, 30]. Our findings support the clinical concept of reducing the administered crystalloids volume to a ratio of 1:1 to avoid any additional detrimental effects on the lungs, which may further contribute to the major postoperative complications [3, 31, 32].

While no significant differences in the systemic hemodynamic parameters were observed between Group 1 and Group 3, the magnitude of the significant decrease in MAP was similar in both Groups 1 and 3. This result suggests an equality of 1:1 and 1:3 ratios in terms of systemic hemodynamic effects. In addition, our findings are in line with the goal-oriented therapy concept, which promotes fluid restriction associated with early onset of vasopressors to maintain perfusion pressure [32, 33]. Therefore, comparing the traditional 1:3–1:4 ratios for crystalloids implied by textbooks [5] with a smaller, 1:1 ratio is already able to ensure physiological homeostasis without compromising the respiratory function.

The choice of the replacement crystalloid was based on the current recommendations of favoring balanced crystalloids over normal saline (0.9% NaCl) [26, 27, 34]. The use of balanced solutions results in lower occurrences of hypochloremia and metabolic acidosis [35]. This may be reflected in the maintenance of normal physiological pH in the animals of Group 1 in the present study, as opposed to development of acidosis in a similar earlier protocol by using normal saline [7].

As concerns the changes of Raw and H following fluid resuscitation, the present findings demonstrate similar trends to those observed previously [7]. However, the results of the present study differs from the previous findings for H, with an increase of approximately 15% detected in our present study opposed to as high as ~ 40% when fluid replacement was performed with 1:1 crystalloid [7]. These differences were also consistent with the development of more severe of lung edema. The primary reason for these differences can also be attributed to the use of different crystalloid solutions in the two studies: normal saline in the previous one and a balanced solution (Ringer’s acetate) in the current study. It has been previously reported that higher levels of body surface indexed extravascular lung water (EVLWI) can be detected in a swine model of fluid resuscitation of hemorrhagic shock when normal saline is used as opposed to a balanced crystalloid solution (Ringer’s lactate) [36]. Our results also support that balanced crystalloids are more favorable opposed to normal saline in terms of pulmonary edema formation [27, 34].

A limitation of our results is related to the experimental setting. The total respiratory system impedance data comprises contributions from the pulmonary system and the surrounding tissues (e.g. diaphragm and the chest wall) [13]. This means that the mechanical parameters obtained in the present study may not solely reflect changes in the pulmonary system, particularly for the tissue parameters [13]. We are not aware of any previous studies addressing the mechanical changes of the chest wall following hemodilution, however, no changes were reported in the resistance and elastance of the chest wall following severe oleic acid-induced interstitial edema [37]. Therefore, we believe that the mechanical properties of the chest wall would not change substantially following hemodilution and the changes we detected are mainly of pulmonary origin. However, due to the masking effects of the fairly constant extrapulmonary tissue mechanical compartments, the real pulmonary mechanical changes are expected to be of greater magnitude. Another limitation of our experimental design is related to the lack of injured tissues like in the presence of traumatism, where hemodilution with crystalloids may affect coagulation factors with subsequent rebleeding. However, our primary aim was to investigate intravascular volume expansion mimicking a clinical situation where the coagulation pathway is functional. While our model did not aim at maintaining the colloid-osmotic pressures or the level of coagulation factors, it still represents clinical approaches resulting from continuous occult bleeding and/or application of stable coagulation factors with crystalloids avoiding fresh frozen plasma. Accordingly, our findings do not allow any conclusions regarding volume replacement under trauma conditions. A further limiting factor is related to the lack of direct monitoring of cardiac output as an indicator of optimal organ perfusion. However, the homeostasis remained physiological (Table 1) despite the drop in MAP, which indicated that anaerobic metabolism did not occur.

## Conclusions

In summary, the results of the present study provide evidence for the optimal fluid replacement ratio when considering crystalloids for fluid replacement following blood loss. Accordingly, a ratio of 1:1 is more appropriate than the historical approach since it is able to meet the needs for maintaining homeostasis while avoiding the adverse effects related to fluid extravasation. With the limitations of extrapolating experimental results to clinical practice, our findings may suggest that considering crystalloid fluid replacement in a ratio of 1:1 to compensate continuous occult bleeding maintains appropriate organ perfusion without the adverse consequences of fluid extravasation.

## Abbreviations

ANOVA: Analysis of variances
BL: Baseline
CaO_2_: Arterial oxygen content
CR1–6: Crystalloid replacement steps
EVLWI: Extravascular lung water
G: Tissue damping
H: Tissue elastance
Hb: Hemoglobin
Hct: Hematocrit
HR: Heart rate
Iaw: Airway inertance
MAP: Mean arterial pressure
P_1_: Pressure at the loudspeaker end
P_2_: Pressure at the tracheal end
PaCO_2_: Arterial partial pressures of carbon dioxide
PaO_2_: Arterial partial pressures of oxygen
pH: Arterial pH
Raw: Airway resistance
TBV: Total blood volume
W/D: Wet-to-dry weight ratio
W1–7: Blood withdrawal steps
Zrs: Impedance of the respiratory system
